# Clinical decision making under uncertainty: a bootstrapped counterfactual inference approach

**DOI:** 10.1186/s12911-024-02606-z

**Published:** 2024-09-28

**Authors:** Hang Wu, Wenqi Shi, Anirudh Choudhary, May D. Wang

**Affiliations:** 1 Coulter Department of Biomedical Engineering, Georgia Insitute of Technology, Atlanta, USA; 2 Department of Electrical and Computer Engineering, Georgia Insitute of Technology, Atlanta, USA

**Keywords:** Clinical decision making, Causal inference, Counterfactual machine learning

## Abstract

**Background:**

Learning policies for decision-making, such as recommending treatments in clinical settings, is important for enhancing clinical decision-support systems. However, the challenge lies in accurately evaluating and optimizing these policies for maximum efficacy. This paper addresses this gap by focusing on two key aspects of policy learning: evaluation and optimization.

**Method:**

We develop counterfactual policy learning algorithms for practical clinical applications to suggest viable treatment for patients. We first design a bootstrap method for counterfactual assessment and enhancement of policies, aiming to diminish uncertainty in clinical decisions. Building on this, we introduce an innovative adversarial learning algorithm, inspired by bootstrap principles, to further advance policy optimization.

**Results:**

The efficacy of our algorithms was validated using both semi-synthetic and real-world clinical datasets. Our method outperforms baseline algorithms, reducing the variance in policy evaluation by 30% and the error rate by 25%. In policy optimization, it enhances the reward by 1% to 3%, highlighting the practical value of our approach in clinical decision-making.

**Conclusion:**

This study demonstrates the effectiveness of combining bootstrap and adversarial learning techniques in policy learning for clinical decision support. It not only enhances the accuracy and reliability of policy evaluation and optimization but also paves avenues for leveraging advanced counterfactual machine learning in healthcare.

## Introduction

Developing Clinical Decision Support Systems (CDSS) that include both diagnosing disease states of patients and formulating corresponding treatment plans is a crucial stride towards realizing the potential of personalized medicine (PM) [1, 2]. However, one of the most significant modeling challenges in applying machine learning to clinical decision-making is its inherent counterfactual nature. When considering treatment options for a patient at any given time point, several alternatives exist. Yet, we only witness the outcome of the treatment option selected by the clinician, leaving the potential effectiveness of other treatments unexplored. To understand the effectiveness of our treatment suggestions, we must compare the counterfactual outcome-what might have happened with a different treatment-with the observed factual outcome.

To solve the challenge of counterfactual inference in clinical decision-making, a traditional approach is randomized controlled trials (RCTs). In these trials, patients are randomly assigned to different treatment groups. By comparing the average outcomes of the treatment and control groups, we can derive a reliable estimation of the treatment effectiveness. However, RCTs have inherent limitations. To adequately capture patient diversity and ensure broad representativeness, these trials need to be conducted on a large scale. Even so, they often provide limited insights into the suitability of treatments for individual patients due to their generalized nature [3]. Most clinical treatment data is available as observational data (e.g., electronic health records (EHRs)) maintained by healthcare providers, hospitals, and insurance companies. In this context, treatments are determined by physicians based on their expertise and patient conditions. Most policy learning algorithms in reinforcement learning require real-time interaction with the environment (patients, in this case) for feedback and updates. Such interaction poses risks, as learning algorithms could make arbitrary decisions potentially detrimental to patient health. Directly deploying these algorithms in a clinical setting would be unethical. Therefore, we focus on learning decision policies offline using EHRs under counterfactual settings.

In clinical decision-making, collecting a sufficient amount of pretraining data presents significant challenges due to personal health data protection regulations and individual privacy concerns. A common strategy to address data scarcity is to aggregate smaller datasets from various data sources (e.g., hospitals). However, this approach leads to high heterogeneity in multicenter datasets owing to differences in clinical protocols and medical devices. This heterogeneity introduces uncertainty, resulting in considerable variability in patient-specific model predictions and decisions [4]. Additionally, even datasets from a single institution exhibit diversity in patient demographics. The challenge of uncertainty in clinical decision-making models is further compounded due to the limited understanding of the clinician’s intrinsic model for selecting treatments and their corresponding reward functions. Neural network-based predictive models, especially in scenarios with limited data, are known to be susceptible to model uncertainty. Policy learning methods relying on estimating clinicians’ action propensity scores to derive optimal policies are also vulnerable to uncertainty. It is observed that models with similar performance levels can exhibit significant disparities in their predictions, particularly in data-scarce cases. Prior research has demonstrated that effectively capturing model uncertainty leads to reduced variance and enhanced exploration in policy learning [4, 5]. Given the critical need for lower risk and higher confidence in medical decision-making, there is an urgent need to address model uncertainty in the estimation of propensity scores and to integrate uncertainty into existing off-policy learning frameworks.

To solve these challenges, we advocate a counterfactual inference framework for learning clinical decision-making from observation data under uncertainty. We first formulate a clinical decision-making process in the framework of contextual bandit. At each round, we will select a clinical action according to the policy and the patient state. Via executing the clinical action, we collect observations on the reward function. Thus, we can collect an observation dataset under a behavioral policy (e.g., the decision made by one physician) $h_0$ is in the format of $\mathcal {D}={(x_i,a_i,r_i)}_{i=1}^{n}$, consisting of patient state $x_i$, clinical action $a_i$, and the observed reward $r_i$. *R* is the accumulated reward by the observed reward at each step. The two tasks in policy learning given the observation dataset $\mathcal {D}$ are: 1) **Evaluation**: For another policy *h*, what is the expected reward *R*(*h*) if it had been applied to the data? 2) **Optimization**: Is it possible to identify a policy *h* parametrized with parameters $\theta$ that maximizes the reward on this dataset, i.e., $h_\theta = \arg \max R(h_\theta )$?

In this paper, we introduce a novel methodology for making informed treatment suggestions with the following contributions to the field:

- Acknowledging the high uncertainty in medical data and the critical need for lower risk in medical decision-making, we propose a bootstrapping-based approach for learning decision-making policies. This method provides not only reward estimates but also confidence intervals, enabling physicians to select actions with reduced variance when necessary.
- Our bootstrapping framework effectively addresses the model uncertainty typically associated with IPS-based estimators, leading to decreased variance in policy evaluation and enhanced policy optimization.
- We address model uncertainty from the perspective of distributionally robust counterfactual risk minimization. Specifically, we introduce an adversarial IPS learner (IPS$_{adv}$), designed to maximize rewards under the worst-case propensity model within a defined uncertainty set.
- We validate the effectiveness of our proposed frameworks (IPS*inv*, IPS*avg*, IPS$_{adv}$) in a clinical scenario involving the oral dosing of anticoagulants, heparin and warfarin. Our approaches not only facilitate better initial dosing policies but also achieve higher rewards. Moreover, we introduce the generation of semi-synthetic and real-world clinical bandit datasets to promote further research in this field.

## Related work

### Treatment recommendation

Effective treatment recommendations are an important component in building CDSS to enhance long-term patient benefits. These recommendations in clinical treatment broadly fall into two categories. **Predictive Modeling**: The first category concentrates on improving the accuracy of future patient outcome predictions, often known as prognosis prediction. This approach is particularly useful in areas like cancer treatment [6] and dermatology [7]. Methods in this category typically employ supervised learning on historical patient data to forecast disease progression, survival outcomes, and specific clinical events following a treatment. The ultimate goal is to suggest treatment options that are likely to yield the best outcomes for the patient. **Reward-Based Policy Learning**. The second category involves developing models that directly link observed clinical features to treatment actions, aiming to maximize overall rewards closely tied to patient health. This approach has been increasingly adopted in recent biomedical studies, using bandit and reinforcement learning algorithms to recommend adaptive treatment policies, particularly in chronic disease and critical care scenarios. Clinical applications include optimizing antiretroviral therapy in HIV patients [8], tailoring anti-epilepsy drugs [9], managing ventilation support in ICU settings [10], and determining optimal antibiotic dosing for sepsis [11]. Contrary to the predictive models of the first group, which align closely with clinician judgment, this category explores alternative optimal actions to create policies that enhance the likelihood of favorable clinical outcomes. This adds complexity as the policy output influences not only the patient’s future health but also subsequent treatment plans. Our algorithm seeks to integrate and build upon the complementary aspects of these approaches, addressing the complexities inherent in clinical decision-making.

### Counterfactual inference

Counterfactual inference includes two basic questions: **counterfactual evaluation** and **counterfactual learning**, also referred to as off-policy learning in the bandit and reinforcement learning literature. The off-policy evaluation aims to estimate the quality of an alternate target policy *h* by assessing its expected reward if applied to the dataset *D*:

1 $$\begin{aligned} \hat{R}_h = \mathbb {E}_h[r] = \sum \limits _{i=1}^n \mathbb {E}_{a \sim h(.|x_i)} \mathbb {E}_{r \sim \mathcal {F}(\cdot |a_i,x_i)}[r]. \end{aligned}$$

Various statistical approaches have been developed to evaluate the quality of target policies based on historical data. There are primarily two classes of evaluation approaches: 1) the direct method (DM) based estimator, also known as regression adjustment, and 2) the importance sampling-based estimator. The Direct method uses a regression approach to fit a parametric or nonparametric approximation to the true reward function as $\hat{r}(x, a; \theta )$, and the reward of a new policy *h* is estimated as:

2 $$\begin{aligned} \hat{R}^{DM}_h = \frac{1}{n} \sum \limits _{i=1}^N \sum \limits _{a \sim h} p_h(a|x_i) \hat{r}(x_i, a), \end{aligned}$$

where $p_h$, known as the propensity score, represents the probability of selecting action *a* under policy *h*, given the observed features *x*. While this approach is straightforward in design, it is susceptible to several biases. The first bias may arise from potential mis-specifications in the reward function $\hat{r}$ (e.g., linear vs. nonlinear models). The second bias emanates from the sampling distribution: the target policy might choose actions differently compared to the logging policy $h_0$. If $h_0$ is biased towards a specific region in the action space, the logged data will predominantly consist of samples from that region, leading to an imbalanced dataset and, subsequently, biased estimation in the reward function.

A common approach to correct for the mismatch in the action distributions under *h* and $h_0$ is importance weights, defined as $w(x, a)= \frac{p_h(a|x)}{p_{h_0}(a|x)}$, where $p_h$ and $p_{h_0}$ are the probability of selecting the action *a* given the observed features, under policies *h* and $h_0$ respectively. Importance sampling-based estimators are built on importance weighting, with a widely popular estimator being the inverse propensity scoring (IPS) estimator [12]:

3 $$\begin{aligned} \hat{R}^{IPS}_h = \frac{1}{n} \sum \limits _{i=1}^N \frac{p_h(a_i|x_i)}{p_{h_0}(a_i|x_i)} r_i, \end{aligned}$$

From the formulation, it can be noted that the IPS estimator is an unbiased estimator of *R*, i.e., $\mathbb {E}\left[\hat{R}^{IPS}_h\right] = R_h$, which makes it well-suited to policy optimization. However, the estimator suffers from high variance in reward estimation, especially when $p_h(a|x)>> p_{h_0}(a|x)$. For consistent estimation, it is standard to assume that whenever $p_h > 0$, then $p_{h_0} > 0$ also, and we assume this throughout our analysis. To reduce the variance of IPS, several techniques have been proposed in the bandit literature. A line of work focuses on regularizing the variance of IPS [13–15] with the POEM estimator being widely used. Another straightforward approach is capping propensity weights [16, 17], which leads to the estimator

4 $$\begin{aligned} IPS^M : \hat{R}_h = \sum \limits _{i=1}^n \frac{p(a_i|x_i)}{max(M, p_{h_0}(a_i | x_i))} r_i; 0< M < 1. \end{aligned}$$

Smaller values of *M* reduce the variance of $\hat{R}_h$ but introduce bias. Given that the IPS estimator is not equivariant [18], thresholding propensity weights exacerbate this effect. Moreover, the IPS estimator is prone to overfitting propensity weights, i.e., for positive reward, policies that avoid actions in the dataset *D* are selected; for negative reward, policies that overrepresent actions in *D* are selected. Hence, Swaminathan and Joachims [18] proposed the self-normalized inverse propensity scoring (SNIPS) estimators, which use weight normalization to counter the propensity overfitting problem of IPS.

5 $$\begin{aligned} SNIPS : \hat{R}_h = \frac{\sum \limits _{i=1}^n r_i w_i}{\sum \limits _{i=1}^N w_i} \text { with } w_i = \frac{p(a_i|x_i)}{p_{h_0}(a_i|x_i)}. \end{aligned}$$

SNIPS has a lower variance than the vanilla IPS estimator because of its ability to normalize and bound the propensity weights between 0 and 1. Additionally, another line of work focuses on reducing both the bias and variance of off-policy estimators by combining the direct method and IPS-based methods in a linear fashion, leading to the doubly-robust estimator [19].

In clinical settings, the behavior policy is typically unknown. Since IPS-based approaches require the behavior policy’s propensity score $p_{h_0}$, we need to impute these scores using a behavior propensity model. The model must accurately represent the clinician’s treatment action probability distribution. If the behavior policy is misestimated, IPS-based estimators suffer from significant bias and variance. Given that we do not know the parametric class of behavior policy, we can leverage universal function approximators such as neural networks to estimate the propensity scores. Neural networks often lead to a reduced approximation error with an increasing number of layers and neurons and have been shown to work well in off-policy bandit scenarios [18, 20]. However, learning a highly accurate model for imputing behavior policy is not enough; our model should provide well-calibrated probability estimates representing true probabilities. Using overparameterized approximators such as neural networks, which are capable of expressing a wide range of functions, along with the limited size and heterogeneity of clinical datasets, leads to model uncertainty i.e., uncertainty regarding the true underlying parameters. Multiple neural networks can achieve similar accuracy. However, the probability estimates can widely differ, and every model might not be able to capture the true conditional probability of the clinician’s actions.

Therefore, the question we ask here is: *How can we confidently estimate the propensity score in the presence of model uncertainty due to the limited scale and heterogeneity of clinical data?* Our algorithms work as a metaframework that can combine existing algorithms.

## Preliminaries

### Problem definition

In this study, we focus on two problems (Table 1) in CDSS: For a patient with observed features *x*, (1) a *predictive modeling* *f* predicts the interested outcome $\hat{y}$ (e.g., diagnosis) that aligns closely with a human physician’s judgment *y*; (2) while *decision-making* learns a policy *h*(*a*|*x*) that suggests the action *a* to take a based on the observed feature *x*, and the objective of the policy is to maximize a specific reward function *r*(*x*, *a*), such as the clinical risk score.

**Table 1:** Two main problems addressed in Clinical Decision Support Systems (CDSSs): Given a patient, we denote the feature vector as *x*, label as *y*, and action as *a*; two problems include (1) a predictive modeling task *f* predicts the outcome $\hat{y}$ aligning closely with a human physician’s judgment *y*; (2) a decision-making task that maximizes a specific reward function *r*(*x*, *a*)

Predictive Modeling	$f: x \rightarrow \hat{y}$	$\hat{y}$ and *y* be as close as possible
Decision-Making	$h: x \rightarrow a$	*a* achieves maximum reward

### Contextual bandit-based clinical decision making

At each round t, we observe a patient with feature representation $x_t$; we choose a clinical action $a_t$ based on some policy ; we observe the outcome/reward $r_t$ of the action we choose, but not for other unchosen actions $a' \ne a_t$. Here $r_t$ may be dependent on $x_t$ and $a_t$, and we may write generically as $r_t=r_t (x_t,a_t)$, for some reward function $r_t$. The goal of policy learning for decision-making is to find the optimal policy *h* that obtains the maximum cumulative reward when applied, i.e., $h^* \sim \arg \max _{h} R = \mathbb {E}_{x_t, a_t \sim h} [r_t]$.

### Uncertainty of predictive models

There are two types of uncertainty in machine learning and deep learning models: **data uncertainty** and **model uncertainty** [21]. Consider a binary classification setting in which we have $y \sim \text {Bernoulli}(\lambda )$, where *y* is the binary classification target, and $\lambda (\cdot |x;\theta )$ is the logit representing the conditional distribution $p(y|x; \theta )$ with feature *x* and parameters $\theta$. In data uncertainty, the logit $\lambda$ is a deterministic function of *x* and $\theta$, i.e., $\lambda = g(x, \theta )$, and the uncertainty in data is reflected in the feature *x*. This uncertainty might be due to inherent noise in the process that generated the data or unaccounted factors that created variability in the targets. This is often referred to as irreducible or aleatoric uncertainty.

On the other hand, model or epistemic uncertainty refers to the uncertainty in the values of the parameters $\theta$ for modeling the prediction, i.e., we are unable to properly constrain our model’s parameters. More specifically, we can model $\lambda$ as a distribution over plausible values instead of a point estimate, as $\lambda \sim \mathcal {P}(\lambda | x, w)$ and are unsure which distributions better explain the data. This could be due to the use of a complex model relative to the amount of training data. Additionally, our choice of model structure might be wrong and is unable to reflect the process that generated the data (here, the clinician). Model uncertainty can be reduced by observing more data; however, typical clinical datasets for bandit learning have limited size ($\le$ 5,000 patients). Our focus here is on tackling model uncertainty caused by uncertainty in the parameters. We explore two popular approaches to quantify model uncertainty in the clinical setting: Model Ensembling and Bayesian Neural Networks.

#### Model ensembling

Deep ensembles proposed by Lakshminarayan et al. [22] is a simple yet powerful method in characterizing the model uncertainty. It has been shown to yield high-quality predictive uncertainty estimates, requires little hyperparameter tuning, and is readily parallelizable. Ensembles tackle uncertainty by collecting predictions from M independently trained deterministic models (ensemble components). We train an ensemble of neural networks (NNs) ($NN_1, ..., NN_M$) by varying the random seed in our training process. The seed affects the initialization of the neural network’s weights and the order of mini-batch samples seen by the neural network during training. At the test time, for a given patient, we output the ensembled action prediction as $p(a|x) = \frac{1}{M} \sum \limits _{m=1}^M p_{NN_m}(a|x)$. In addition, the collection of prediction values $p_{NN_i}(x); i=\{1,2, ..., M\}$ can be seen as samples from the distribution $p(\lambda |x, \theta )$ describing the model uncertainty.

#### Bayesian neural networks

Bayesian inference is a principled approach to modeling the distribution over possible outcomes and estimating the uncertainty in the prediction of a machine learning model. Bayesian Neural Networks (BNNs) are neural networks whose parameters $\theta$ are represented by probability distributions, so the uncertainty of weights characterizes the uncertainty of models. Given a dataset *D* = ${(x_i, y_i)}_{i=1}^{N}$, BNN is defined in terms of a prior *p*(*w*) on the weights and the data likelihood *p*(*D*|*w*). By sampling from the posterior weight distributions, BNN could train an infinite number of different realizations of the NNs, and these realizations capture the model uncertainty in the predictive distribution $p(\lambda |x, \theta )$. However, training BNNs is much more challenging since we need to compute the posterior distribution. Various approximate inference methods are proposed to efficiently train BNNs, such as MC-Dropout [23], Variational Inference, [5] and Noisy Natural Gradient method [24]. Bayesian approaches to uncertainty estimation have been proposed to assess the reliability of clinical predictions [4] but have been applied to very few real-world policy learning settings using clinical data.

##### Variational Inference

Variational approximation methods aim to estimate the weight posterior by maximizing the evidence lower bound (ELBO) to fit an approximate posterior $q(w|\theta )$, given data *D*. Variational inference is formulated as an optimization problem of minimizing the Kullback-Leiber (KL) divergence between the approximate *p*(*w*) and exact $q(w|\theta )$ posterior. The loss function embodies a trade-off between data-dependent likelihood cost and prior-dependent complexity cost as follows:

6 $$\begin{aligned} L(D,\theta ) = \mathbb {E}_{q(w|\theta ))}[log(p(D|w)] - KL[q(w|\theta )||p(w)], \end{aligned}$$

where *p*(*w*) is the prior distribution on weights, which enforces simplicity. The most common approach to learn an approximate posterior over the weights $q_{\theta }(w)$ given the prior is mean-field variational inference wherein we assume a fully factorized Gaussian prior and posterior, $q(w) = \prod _{i=1}^{m}q_i(x)$. This reduces the computational complexity of estimating ELBO. To reduce the time complexity of computing KL-divergence during a forward pass through the network, we leverage Monte Carlo estimates. Blundell et al. [5] proposed Bayes-by-Backprop by applying the re-parametrization trick from Kingma et al. [25] to variational inference and reduced the computational complexity involved in calculating the data likelihood expectation $E_q[log(p(D|w))]$ over *q*(*w*|*D*). They estimate the variational inference loss function by sampling weights from the posterior *q*(*w*|*D*):

7 $$\begin{aligned} L(D,\theta ) \approx \sum \limits _{i=1}^n log [q(w^i|\theta )] - log P(w^i) - log P(D|w^i), \end{aligned}$$

where $w^i$ are the sampled weights. To enable training by backpropagation, they choose a Gaussian variational posterior on weights given as $q(w|\theta ) = \prod _{i=1}^n \mathcal {N}(w^i|\mu ,\sigma ^2)$. To perform inference using BNNs, Monte Carlo sampling is performed from the weight distribution. Multiple networks are sampled from the variational posterior *q*, and their predictions are averaged to compute the network output. In BNNs learned using variational inference, typically, both the mean and variance of weights are learnable.

##### MC-Dropout

Gal et al. [23] showed that optimizing a standard neural network with dropout and $L_2$ regularization techniques is equivalently a form of variational inference in a probabilistic interpretation. MC-Dropout is quite popular due to the simplicity of the idea: by enabling dropout during testing and applying different dropout masks, multiple networks can be sampled to predict the output and related uncertainty. This contrasts with performing inference using a deterministic neural network wherein the dropout approximation is fixed at the test time. However, in practical applications, MC-Dropout faces challenges, such as the choice of dropout probability and $L_2$ regularization, the position to insert the dropout layers at, etc.

## Bootstrapped counterfactual evaluation

In this section, we first introduce the off-policy evaluation problem and then present our framework for bootstrapped-based evaluation. For each patient with feature *x*, a policy $h_0$ (e.g. a physician) recommended a treatment *a*, and a reward $r=r(x, a)$ was observed. The collection of these triplets $\{ (x_i, a_i, r_i) \}_{i=1, ..., N}$ forms an off-line dataset $\mathcal {D}$. The goal of offline evaluation is defined as follows:

### Definition 1

Given an offline dataset collected from a logging policy $h_0$, for a new policy *h*, we aim to estimate its expected reward *R*(*h*) using the offline dataset $\mathcal {D}$ only.

This problem is important as it represents a majority of scenarios arising during the evaluation of a clinical decision support system. Suppose we use our machine learning algorithm to build a new treatment policy *h* using EHRs obtained from a hospital; how do we ensure this policy is advantageous before deploying it in the clinic? Traditionally, to get an unbiased estimate of *R*(*h*), we can use the inverse propensity scoring estimator as follows:

8 $$\begin{aligned} IPS: R(h) = \sum \limits _{i=1}^N \frac{p_h(a_i|x_i)}{p_{h_0}(a_i | x_i)} r_i, \end{aligned}$$

where $p_h$ is the propensity score of the policy *h*.

In the clinical setting, however, $p_{h_0}(a|x)$ is not available, as physicians will not record the exact probability of them choosing a treatment. Modeling $h_0$ via supervised learning using a maximum likelihood-based approach is possible but introduces additional model-uncertainty: *There can be multiple versions of* $h_0$ *that share equal probabilities and evaluate the same on a finite training set of* *N* *data points, however having different behaviors on other data points (test set).* To see this, imagine our policy is only a polynomial of degree ‘$N+1$’, and with *N* data points *x*, we can fit an infinite number of functions *f*(*x*, *w*) attaining zero error and satisfying the learning objective, thus, giving out a diverse range of model parameters *w*. The distribution over the model parameters $w \sim p(w)$ induces uncertainty in the learned function, characterized by $\hat{h_0} \sim U(f_w)$, subsequently leading to variance in the marginalized predictive probability distribution $p_{h_0}(a|x)$. Considering more complex functions such as neural networks, the potential solutions for $h_0$ are even more.

Thus, we propose to reduce such model uncertainty in IPS-based estimators using a bootstrapping-based approach. By bootstrapping over multiple resamples of the dataset *D* and using model ensembling, we can reduce the uncertainty from learning $h_0$ and obtain a better estimate of the policy reward. In addition, we also obtain a confidence interval for the overall performance of the new policy *h*. When we have multiple policies, we can choose not only based on the mean rewards but also the tightness of the reward confidence interval as a criterion for the stability of the policy. We present our bootstrapped policy evaluation framework in Algorithm 1.

**Figure:**
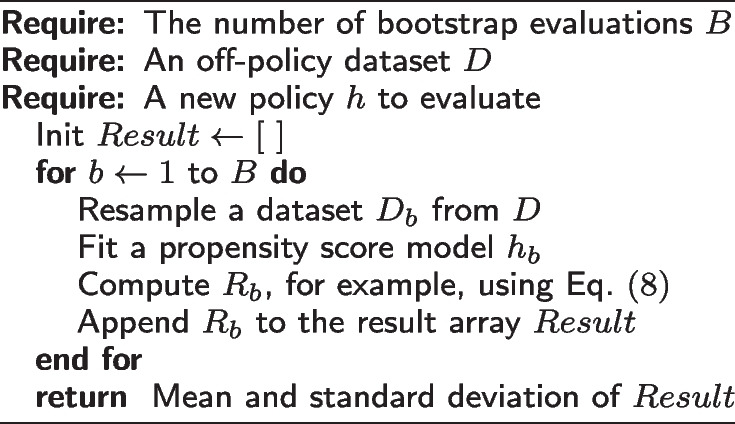
**Algorithm 1** Bootstrapped Policy Evaluation

We explore deterministic NN ensembles and probabilistic BNN-based approaches to tackle model uncertainty. For simplicity, we discretize the clinician actions *a* and formulate the propensity score imputation problem as a multiclass classification problem. We train a classifier on $(x_i,a_i) \in D$ and derive the propensity scores from softmax-layer probability scores.

After bootstrapping *B* networks, we propose our counterfactual estimators based on the propensity score estimates obtained from those models:

9 $$\begin{aligned} \textit{IPS}_{avg}: \hat{R}(h) = \frac{1}{N}\sum \limits ^N_{i=1}\frac{p_h(a|x)}{\frac{1}{M}\sum ^M_{m=1}p^m_{h_0}(a|x)}, \end{aligned}$$

10 $$\begin{aligned} \textit{IPS}_{inv}: \hat{R}(h) = \frac{1}{N} \frac{1}{M}\sum \limits ^N_{i=1} \sum \limits ^M_{m=1}\frac{p_h(a|x)}{p^m_{h_0}(a|x)}, \end{aligned}$$

where $p^b_{h_0}$ is the propensity score derived from $b^{th}$ bootstrapped model.

The simplest approach is to average the propensity scores from bootstrapped models to reduce the variance of $p_{h_0}$ leading to IPS$_{avg}$. The inverse estimator, IPS$_{inv}$, computes a harmonic mean of propensity scores and is equivalent to averaging the estimated rewards $\hat{R}(h)^m$ from each bootstrapped model. The average estimator can also be seen as a special case of multiple importance sampling and is equivalent to the Balance Heuristic estimator (Veach et al. [26]) when $N=KN_k$:

11 $$\begin{aligned} \hat{R}=\sum \limits _{k=1}^K\sum \limits _{i=1}^N\frac{p(x_{ik})}{\sum _{j=1}^{K}N_jp_0^j(x_{ik})}r_i;\ where\ \sum \limits _{k=1}^KN_k=N. \end{aligned}$$

By bootstrapping over multiple resamples of the dataset *D*, we can reduce the uncertainty from learning $h_b$ and obtain a better estimate of the policy reward. In addition, we also obtain a confidence interval for the overall performance of new policy *h*. In this way, when we have multiple policies, we can choose based not only on the average reward but also on the tightness of the confidence intervals as an evaluation of the stability of the policies.

## Bootstrapped counterfactual learning

The goal of counterfactual learning is

### Definition 2

Given an off-line dataset collected from a logging policy $h_0$, we aim to estimate find $h^*$ using the off-line dataset $\mathcal {D}$ only, such that its expected reward *R*(*h*) is maximized, i.e.

$$\begin{aligned} h^* = \arg \max R(h) \end{aligned}$$

In the previous section, we have developed how to evaluate any *h* for its expected reward *R*(*h*). Intuitively, if we have a finite selection of *h*, by an exhaustive evaluation of all *h*, we can find the optimum $h^*$. For an infinite space of *h*, we can apply gradient-based optimization; we can evaluate the gradient as

12 $$\begin{aligned} \nabla R(h) = \sum \limits _{i=1}^N \frac{\nabla h(a = a_i|x_i)}{h_0(a = a_i | x_i)} r_i \end{aligned}$$

When we parametrize *h* with deep neural networks, we can automatically compute its gradient via back-propagation techniques to apply gradient-based optimization. Based on our bootstrapped evaluation algorithm, we design a bootstrapped learning algorithm as follows:

**Figure:**
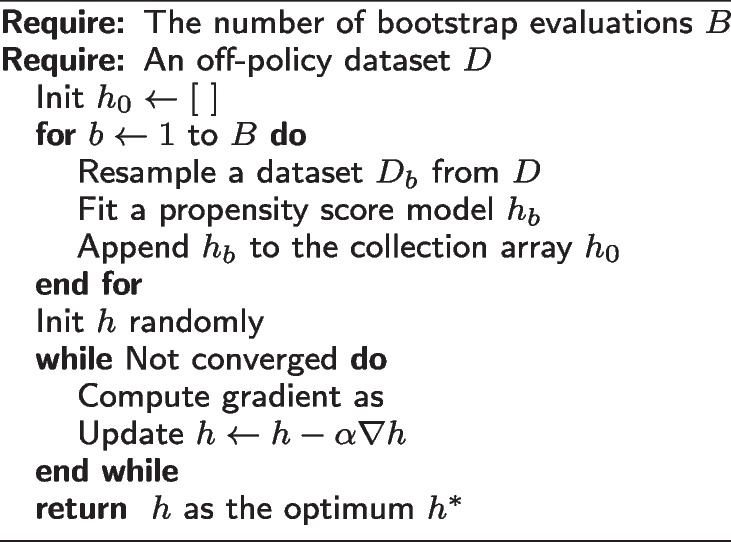
**Algorithm 2** Bootstrapped Policy Optimization

The benefit of this algorithm is that by adding bootstrapping, we reduce the variance of learning $h_0$ to improve the performance and stability of the learned *h*. In addition, we can also populate the confidence intervals.

## Adversarial bandit learner

In decision theory, robust decision-making based on Wald’s maximin paradigm [27] suggests acting pessimistically - the optimal decision is one with the least bad worst outcome. Since multiple propensity scoring models are likely and bootstrapping optimizes the learned policy against an ensemble, the empirical reward $\hat{R}[h_w]$ cannot be used as a performance certificate for the optimal true reward. This is because we are not explicitly tackling the uncertainty due to the worst-case propensity model $h^{worst}_0$. To address this limitation, we propose an adversarial learning-based framework, treating the propensity model parameter $P(\theta )$ distribution with skepticism and optimizing the worst-case reward objective with respect to pessimistic model parameters. Instead of selecting the worst-case model from the ensemble, we assume that model parameter distribution belongs to an uncertainty set $U_{\epsilon }(P)$, which is already constrained by the cross-entropy loss by virtue of behavior policy imputation (the goal of $h_0$ is to model clinician’s actions accurately). Hence, we can derive an adversarial robust counterfactual learning objective as follows:

$$\begin{aligned} \textit{IPS}_{adv}: & h^*_w \sim \arg \max _w \min _{w_0} R(h,h_0) + \lambda CE(\hat{h}_0, h_0), \\ R(h,h_0) & = \frac{1}{N} \sum \limits _{i=1}^n \frac{p_h(a|x; w)}{p_{\hat{h}_0}(a|x; w_0)} r_i, \\ CE(\hat{a}_i, a_i) & = -\sum \limits _k a_k log\hat{a}_k \text { ; } \hat{a}_k = \hat{h}_0(x_k;w_0), \end{aligned}$$

where *CE* is the standard multiclass cross-entropy loss, $\lambda$ is a hyperparameter defining the trade-off between accurate behavior policy imputation (2$^{nd}$ term) vs reward maximization (1$^{st}$ term). Consequently, we propose an adversarial policy learning framework (IPS$_{adv}$) (Algorithm 3) as an iterative two-player optimization scheme. *h* is optimized to maximize reward against the worst-case possible $h_0$, which acts in an adversarial manner to *h*: the goal of $h_0$ is to learn a classification model to impute clinician’s action probabilities accurately and, at the same time, reduce the reward achieved by learned policy *h*.

**Figure:**
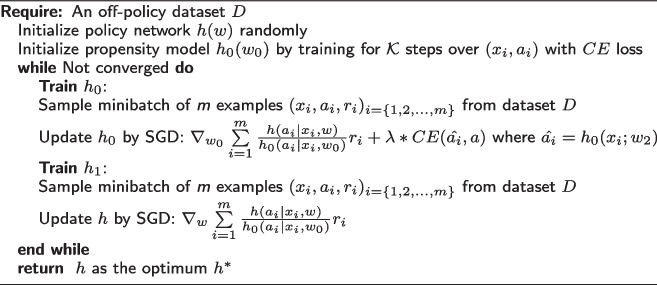
**Algorithm 3** Adversarial Policy Optimization

## Results and discussions

We evaluate the efficacy of our proposed frameworks on the non-clinical semi-synthetic bandit dataset as well as on the clinical task of dosing initialization for orally administered anticoagulant drugs. Anticoagulants are blood thinners administered to remove blood clots, and their dosage during treatment initiation varies significantly across patients. Moreover, incorrect dosing can have significant side effects, thus making it a challenging clinical setting for treatment recommendation systems. We consider two commonly used anticoagulants in hospitals: warfarin and heparin. We use two freely available electronic health records databases to derive the clinical bandit datasets: 1) PharmGKB (Consortium 2009) [28] for warfarin dosing and 2) Multiparameter Intelligent Monitoring in Intensive Care (MIMIC-III v1.4) [29] for heparin dosing. The semi-synthetic dataset is derived from a fully-labeled classification dataset (with access to counterfactuals) by simulating the logging/clinician policy. For Warfarin dosing, we have access to counterfactuals and artificially simulate the logging policy to derive a semi-synthetic dataset. For heparin dosing, we construct a true real-world bandit dataset from MIMIC-III without access to counterfactuals.

### Non-clinical dataset (Semi-synthetic)

We select two multiclass classification datasets from the UCI repository [30], namely Optdigits and Letter, which have been previously used for off-policy bandit evaluation [31, 32] and convert them to contextual bandit dataset by choosing actions derived from a multiclass logistic regression policy trained on 5% of the dataset, similar to Dudik et al. [31].

### Clinical datasets

#### Warfarin dosing (Semi-synthetic)

Using the PharmGKB [33] dataset, we develop a case study to evaluate our framework on warfarin dosing. Warfarin dosing is concerned with determining the correct dosage of the blood anticoagulant drug for a heart patient. The dataset includes patient information (demographics, physiological, and genotype features) with the final ideal therapeutic dosage. Warfarin’s administration needs to be monitored closely since incorrect dosage can lead to adverse side effects such as heart attacks. The therapeutic dosage varies widely across patients due to different contextual features. Physicians typically prescribe an initial dose, adjusted according to the patientś response. Previous work [34] on predicting dosage policies using bandits, discretize the dosage into three categories, ‘low’ (< 21 mg/wk), ‘medium’ ($\ge$ 21 mg/wk, $\le$ 49 mg/wk) or ‘high’ (> 49 mg/wk). Although, recently, warfarin dosing has been approached in the continuous domain, which allows for finer adjustments, we focus on tackling uncertainty in propensity score estimation under a discrete dosage setting to keep the overall formulation simple. With dosage discretization, the Warfarin dataset was converted to a supervised classification dataset $D = {(x_i,y_i)}_{n_i=1}$ with access to treatment counterfactuals. This provides us with the ground-truth action for each patient. Since the dataset is supervised, we simulate a contextual bandit environment by simulating the clinician’s policy and using a custom reward function. We follow the Supervised $\rightarrow$ Bandit conversion approach highlighted in [13, 19, 35] and simulate expert (clinicianś) behavior using stochastic logging policy to sample $y^*_i = h(*|x_i)$ with reward defined based on the match between ground-truth and sampled actions, $r_i = I(y_i = y^*_i)$. We simulate the following stochastic logging policies with 3 and 5 discrete dosage levels (policy actions). These policies are also referred to as expert policies.

1. LR: We follow the experimental design specified in [13] and use a multiclass logistic regression model trained on 5% data as logging policy. For different simulations, we randomly sample 5% data from our training set and fit a multiclass logistic regression model to obtain weight vector $w_{lr}$. To introduce further stochasticity, we randomly perturb $w_{lr}$ using random noise drawn from a standard normal distribution $u \sim N[0,1]$.
2. PHARMA: We adopt the clinical policies (WPGA, WCGA) [33] as our base deterministic policies ($h_1, h_2$). Both WPGA and WCGA are clinically motivated linear models, with WPGA incorporating genotype features to improve over WCGA. Our aim was to emulate clinicians using WPGA or WCGA for dosage recommendation and combine them in a stochastic manner. Motivated by the friendly softening approach proposed by Farajtabar et al. [35], we transform the deterministic policy into stochastic policy by drawing actions $a_i = h_0(x_i)$ from a mixture of these models with equal probability.
  13 $$\begin{aligned} a_i = \left\{ \begin{array}{ll} h_1(x_i),\quad r_i <= 0.5 \\ h_2(x_i),\quad otherwise \end{array}\right. \end{aligned}$$
  where $r_i \in [0,1]$ is a random number for patient $x_i$.

#### Heparin dosing (True bandit)

Heparin is one of the most commonly used anticoagulant medications in hospitals and ICUs. The dosage of intravenous unfractionated heparin is commonly based on the patient’s weight, as per most clinical practice guidelines [36]. Such a weight-based approach alone may result in improper dosage for obese patients. Although some works have recommended using an adjusted body weight [37], in practice, activated partial thromboplastin time (aPTT) is a good indicator of blood coagulation level. There is significant variation in the guidelines for the initial loading dose of heparin, the rate of dosage, and the time measurement intervals of aPTT. A higher aPTT level reflects slow blood clotting, whereas a low level indicates fast clotting. Blood samples are usually taken every 4-6 hours to measure the levels of aPTT, and the result of anticoagulation therapy is analyzed by observing whether aPTT reaches the therapeutic window timely. Typically, aPTT between 60s and 100s is considered therapeutic, with aPTT > 100s being supra-therapeutic and aPTT < 60s being subtherapeutic. While machine learning techniques have tried to develop the ability to provide clinical decision support for heparin dosing, the high patient variability has led to the underperformance of multinomial logistic regression-based models [38]. Here, we formulate heparin dosing as an offline bandit problem by considering the aPTT after 6 hours of dosage initialization as the reward outcome. We discretize the Heparin dosages into 3 categories(actions) ‘low’(< 10 mg/wk), ‘medium’ ($\ge$ 10 mg/wk, $\le$ 15 mg/wk) or ‘high’ (> 15 mg/wk). The outcome of interest was the aPTT value 6 hours after the initial heparin infusion, and the rewards were defined as:

14 $$\begin{aligned} r_i = \left\{ \begin{array}{ll} 1,\quad 60s<= aPTT_{t=6 hrs} <= 100s \\ 0,\quad otherwise \end{array}\right. \end{aligned}$$

Patient demographics and physiological features of interest used to define the context included: age, height, weight, ethnicity, gender, obesity, creatinine concentration, SOFA score, type of ICU admission, end-stage-renal-disease (ESRD), and pulmonary embolism. These features contribute collectively to the patient’s response to the Heparin dose; for instance, creatinine concentration reflects the filtration function of glomeruli and, together with ESRD, serves as an indicator of renal function. We selected these features in line with the previous studies [38, 39], with most of the features being statistically significant for predicting aPTT outcomes. To create the patient cohort, we follow the scheme proposed by Ghassemi et al. [38]. A total of 4,761 adult patients who had undergone heparin dosing during their ICU stay were extracted from the MIMIC-III database. We included only those patients with aPTT measurements 6 hours after the initial Heparin infusion, reducing the cohort size to 2,981. Further, some patients had missing covariates, and by removing these patients, we obtained 2,136 patients. Lastly, we removed patients who were transferred from another hospital since their Heparin infusion might have started before the ICU admission, and we have limited knowledge of medical interventions taken before transfer. Our final cohort comprises of 1,378 patients.

### Baselines

We consider two popular off-policy estimators, IPS & SNIPS, and use the propensity score imputed from a single neural network with vanilla IPS/SNIPS formulation as the baseline. The single neural network can be a deterministic neural network in the case of an NN ensemble or a network obtained by sampling once from the posterior weight distribution of Bayesian NN.

Our logging policy imputation model is a single hidden-layer perceptron network with ReLU activation units. We establish baseline estimators by selecting one of the bootstrapped models as a propensity score estimator. We denote these baseline estimators as Vanilla SNIPS/Vanilla IPS. To bootstrap the deterministic NN model, we randomly initialize the model weights and use dropout (0.25) to fit the models. To train BNN with variational inference framework, we follow the ‘Bayes-by-Backpropagation’ approach [5] assuming a scale mixture of two Gaussian densities as the prior distribution for weights $w_{h_0} \sim 0.5N(0,0.5) + 0.5N(0,0.002)$. The network configurations differ for Warfarin dosing (hidden units = 20) and Heparin dosing (hidden units = 40). We use the Adam optimizer [40] ($\beta _{1}$ = 0.999, $\beta _{2}$ = 0.9) with a learning rate of $1e^{-3}$ and mini-batch size of 50 for both datasets, and use progressive validation to detect convergence. We determined the optimal training hyperparameters using 5-fold cross-validation on both datasets. For adversarial IPS learners, we determined $\lambda$ = 1 to be optimal after experimenting with multiple values (0.5, 1, 1.5, 2).

### Policy evaluation

In this experiment, we evaluate whether a bootstrapping-based framework leads to a more confident reward estimation of a custom clinical policy. Here, we leverage the semi-synthetic non-clinical and Warfarin dosing datasets since they allow for the comparison of the estimated policy reward with the ground-truth reward (estimated from counterfactuals). We perform 20 simulations and report the root-mean-squared error (RMSE = $E[\hat{R}(h)-R(h)]^2$) of our proposed estimators and baselines over these 20 sampled datasets, where *R*(*h*) is the ground-truth reward. We follow the following methodology of Dudik et al. [19] during each simulation to derive the semi-synthetic bandit dataset

1. For each logging policy, we create a partially-labeled bandit dataset by applying the transformations described in the previous *Waefarin Dosing (semi-synthetic)* section.
2. We randomly subsample 70% of the synthetic-bandit dataset as our evaluation dataset and divide it into train/validation sets in an 80/20 ratio for fitting the propensity model.
3. We obtain the evaluation policy *h* by training a multiclass logistic regression model on a full classification dataset and define its classification accuracy as the ground truth reward *R*(*h*).
4. We bootstrap 10 models ($\hat{h}_0^b, b \in \{1,2,...10\}$) for imputing logging policy propensity scores as described in the *Bootstrapped Counterfactual Evaluation* section. For the ensemble approach, we initialize 10 models with seeds in multiples of 2. In variational inference, we train 10 BNNs and sample 10 models, one from each of the 10 weight distributions. For the MC-Dropout model, we apply dropout randomly to sample networks during inference. We also resample data while bootstrapping an NN ensemble model or training a new BNN.

### Policy learning

In this experiment, we use the bootstrapping and adversarial learning frameworks to learn optimal policies with maximum reward. Based on the performance of our bootstrapped estimators for policy evaluation, we expect that addressing uncertainty with bootstrapping and adversarial formulations will translate to learning better policies. We perform 10 simulations and learn the dosing policy *h* using IPS-based loss formulation and minibatch stochastic gradient descent. As the choice for learning $\hat{h}_0$ (i.e., using NNs vs using Bayesian NNs) is orthogonal to the policy learning method, in this section, we use regular NN for demonstration. We follow the following steps during each simulation:

1. We randomly split the data into training (70%) and test (30%) sets.
2. For each logging policy type, we obtain a partially-labeled semi-synthetic bandit dataset for Warfarin dosing by applying the transformations described in the previous *Warfarin Dosing (semi-synthetic)* section. Moreover, we also consider the Heparin dosing dataset, which is a true bandit dataset and allows us to evaluate policy learning in a non-simulated real-world clinical setting.
3. Bootstrapping: We bootstrap ten models for imputing the logging policy. By incorporating the average and inverse learning formulations into IPS and SNIPS estimators, we learn optimum policies $h_{avg}$ and $h_{inv}$, respectively.
4. Adversarial Bandit Learner: We train the models $h_0$ and *h* alternately using the IPS$_{adv}$ loss formulation. Before initiating the adversarial training, we initialized the propensity model $h_0$ by training it for four epochs on the bandit dataset. This assures that $h_0$ initializes with parameters not widely different from the optimal propensity model, which stabilizes the subsequent adversarial learning process. We train both networks alternately for 100 epochs with a learning rate of 0.001.

To evaluate our frameworks, we report the mean reward achieved by our learned policies along with their variance ($\mu _{R(h)} \pm \sigma _{R(h)}$). For Warfarin dosing, we execute the learned policy on the test dataset for Warfarin dosing and compare the predicted actions with ground truth dosage actions from the full classification dataset. For Heparin dosing, since we have access to a real-world bandit dataset, we do not have access to counterfactuals, i.e., the optimal ground-truth dosage for each patient. Hence, we leverage the SNIPS estimator for evaluating the performance of our learned policies, given that offline SNIPS estimates are highly correlated to the true (online) performance for a wide range of policies by Zenati et al. [41]. In our evaluation experiments (Table 2), we found SNIPS to have lower variance and bias than IPS.

**Table 2:** Policy Evaluation: Mean Average Error ($\mu$ ± $\sigma$) of self-normalized inverse propensity scoring (SNIPS)-based estimators. Optdigits and Letter are two multiclass classification datasets from the UCI repository [30]. *LR *Logistic Regression, *BNN *Bayesian Neural Network, *NN *Neural Network

Dataset	Expert Policy	SNIPS($h^{true}_0$)	$\hat{h}_0$ - NN			$\hat{h}_0$ - BNN			
			Vanilla SNIPS	NN Ensemble		Vanilla SNIPS	BNN (Variational Inf.)		MC-Dropout
				SNIPS$_{inv}$	SNIPS$_{avg}$		SNIPS$_{inv}$	SNIPS$_{avg}$	SNIPS$_{avg}$
UCI	OPTDIGITS (10 actions)	2.6 ± 0.4	38.7 ± 25.2	17.6 ± 6.7	**1.0 ± 0.2**	5.5 ± 2.8	11.2 ± 4.1	1.1 ± 0.7	**0.2 ± 0.1**
	LETTER (26 actions)	21.7 ± 0.7	14.3 ± 0.8	14.1 ± 0.5	**12.4 ± 0.3**	34.8 ± 5.4	37.0 ± 0.9	32.1 ± 1.0	**2.8 ± 0.5**
Warfarin	LR (3 actions)	6.9 ± 0.9	15.6 ± 18.7	9.0 ± 2.6	**7.8 ± 0.8**	24.3 ± 19.9	26.1 ± 6.8	**7.5 ± 0.9**	**7.0 ± 0.7**
	LR (5 actions)	10.0 ± 0.6	11.6 ± 6.7	9.3 ± 2.3	**10.1 ± 0.9**	12.7 ± 11.2	13.3 ± 5.4	**9.3 ± 4.5**	**10.3 ± 0.6**
	PHARMA (3 actions)	20.6 ± 3.2	20.9 ± 18.7	17.5 ± 4.6	**15.3 ± 1.1**	19.0 ± 12.4	17.9 ± 4.1	**15.1 ± 1.3**	**12.0 ± 0.7**
	PHARMA (5 actions)	11.5 ± 1.3	12.8 ± 5.0	13.6 ± 3.5	**12.5 ± 1.1**	9.6 ± 5.2	6.7 ± 4.2	**8.0 ± 2.2**	**11.6 ± 0.6**

### Main results

We present the policy evaluation results for SNIPS and IPS estimators on the Warfarin bandit dataset (LR and PHARMA policies) in Tables 2 and 3 respectively. We highlight both bias and variance of the estimated policy rewards. Using bootstrapping leads to significantly lower bias and variance, even in the case of SNIPS, which typically has lower variance due to weight normalization. Comparing the two bootstrap-based estimators, we find that *average* propensity score estimator can achieve lower policy evaluation bias compared to the *inverse* estimator. We also observe that NN ensemble and MC-Dropout-based networks lead to slightly better variance reduction compared to BNNs, which is in line with the uncertainty reduction results observed in [22].

**Table 3:** Policy Evaluation: Mean Average Error ($\mu$ ± $\sigma$) of inverse propensity scoring (IPS)-based estimators. Optdigits and Letter are two multiclass classification datasets from the UCI repository [30]. *LR *Logistic Regression, *BNN *Bayesian Neural Network, *NN *Neural Network

Dataset	Expert Policy	IPS($h^{true}_0$)	$\hat{h}_0$ - NN			$\hat{h}_0$ - BNN			
			Vanilla IPS	NN Ensemble		Vanilla IPS	BNN (Variational Inf.)		MC-Dropout
				IPS$_{inv}$	IPS$_{avg}$		IPS$_{inv}$	IPS$_{avg}$	IPS$_{avg}$
UCI	OPTDIGITS (10 actions)	4.7 ± 0.6	29.1 ± 11.9	21.2 ± 8.9	**3.5 ± 0.4**	8.8 ± 25.4	6.0 ± 4.9	5.7 ± 0.6	**3.2 ± 0.7**
	LETTER (26 actions)	22.9 ± 0.5	2.0 ± 1.5	1.5 ± 1.0	3.9 ± 0.9	23.3 ± 8.2	24.8 ± 2.3	33.1 ± 1.2	516.3 ± 6.8
Warfarin	LR (3 actions)	28.3 ± 1.1	47.6 ± 0.9	46.3 ± 3.6	**47.8 ± 0.8**	228.6 ± 193.4	308.0 ± 79.1	43.6 ± 1.1	**12.5 ± 1.1**
	LR (5 actions)	41.7 ± 0.9	66.4 ± 38.0	56.4 ± 11.9	**62.8 ± 1.0**	824.6 ± 419.2	547.9 ± 148.9	48.0 ± 17.6	**17.8 ± 1.3**
	PHARMA (3 actions)	16.5 ± 1.8	13.7 ± 9.1	17.3 ± 5.0	**20.7 ± 1.2**	13.6 ± 4.8	43.9 ± 21.2	17.1 ± 2.4	**4.0 ± 1.5**
	PHARMA (5 actions)	11.4 ± 2.7	14.3 ± 6.2	11.0 ± 5.1	**19.7 ± 2.6**	58.6 ± 111.6	13.0 ± 8.3	15.0 ± 9.2	**12.1 ± 1.1**

### Impact of number of bootstraps on evaluation error

**Fig. 1:**
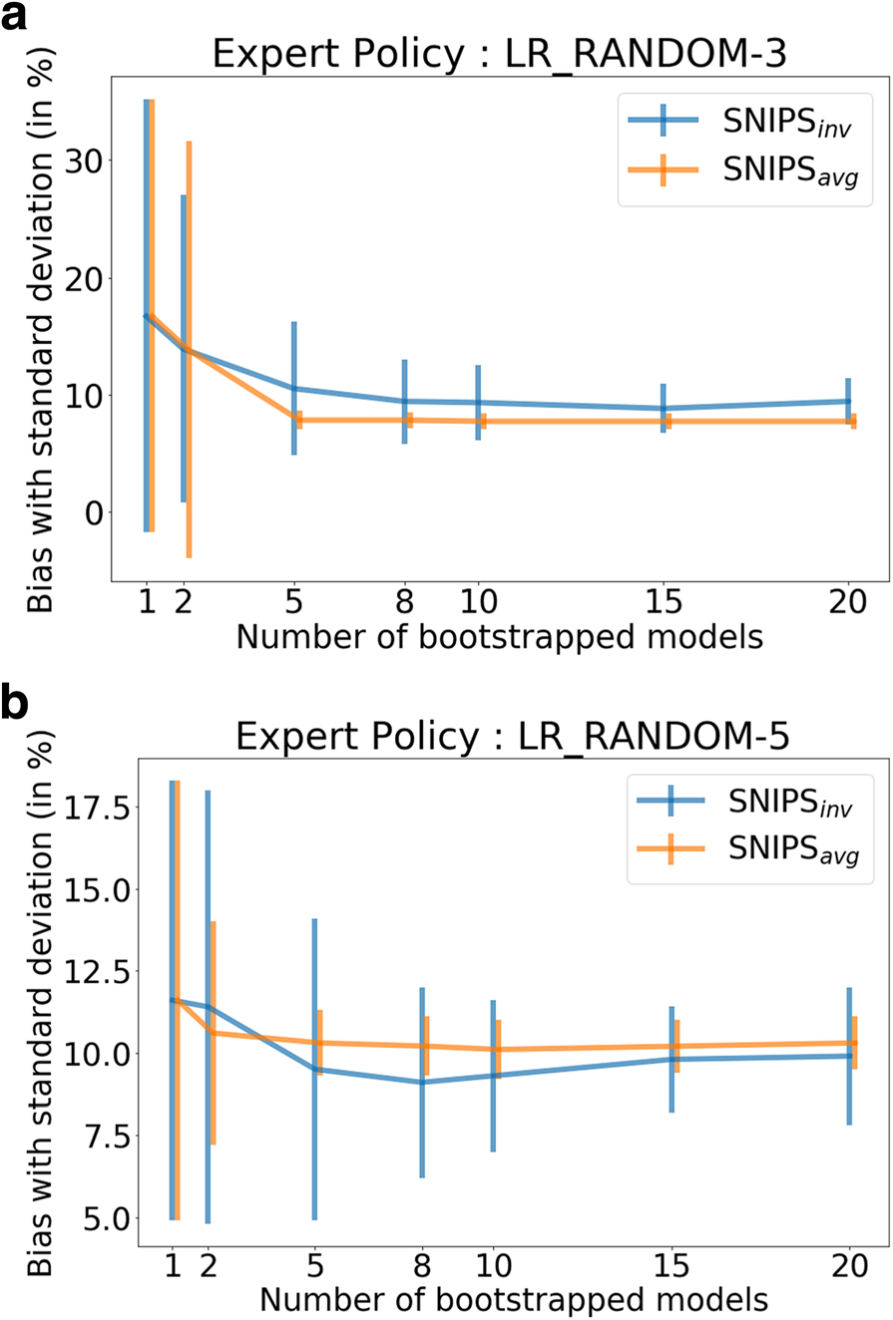
Policy Evaluation: Impact of bootstrap count on mean average error and standard deviation of reward estimates ($\hat{h}_{NN}$)

In the case of the NN ensemble, we also evaluate the impact of bootstrap count on the reduction in bias and variance of SNIPS-based reward estimators (Fig. 1). We observe that an ensemble of 5 neural networks performs sufficiently well in reducing both the variance and bias. As the number of bootstrapped models increases, the bias and variance of SNIPS$_{inv}$ and SNIPS$_{avg}$ estimators reduce significantly with SNIPS$_{avg}$ achieving lower bias and variance. Thus, bootstrapping multiple models allows for sampling from multiple proposal distributions and avoids the situation wherein a single propensity score model suffers from very low probability coverage over certain regions of the action space.

**Fig. 2:**
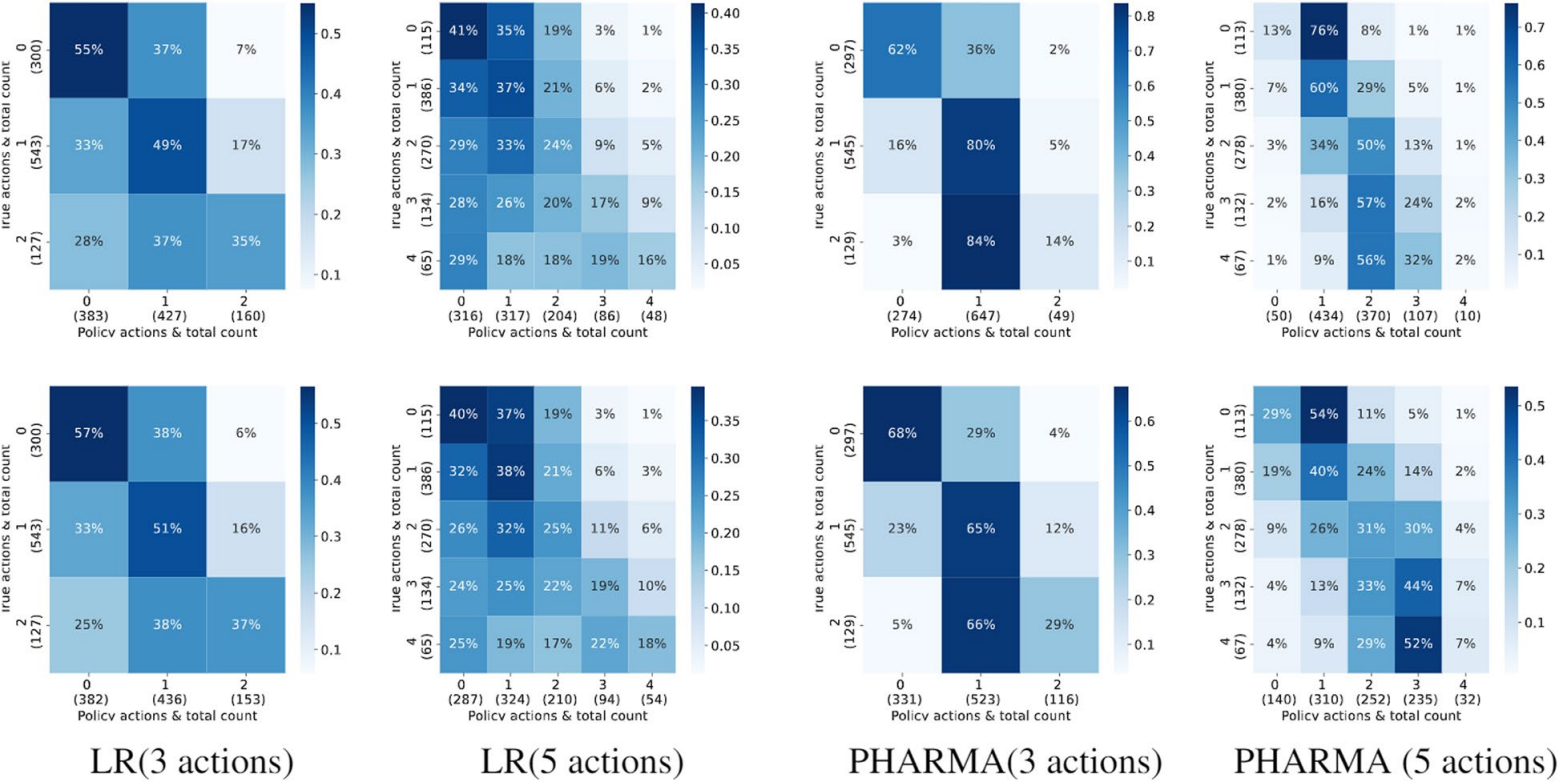
Policy Learning: Comparison of true clinical actions and policy actions for Vanilla inverse propensity scoring (IPS) (1$^{st}$ row) and IPS $_{inv}$ (2$^{nd}$ row). Each cell represents the predicted count as a percentage of the true action total count

In Tables 4 and 5, we highlight the results of policy learning on clinical datasets. Using bootstrapping leads to improved policy learning both on semi-synthetic data (LR logging policy in Warfarin dosing) as well as true-bandit data (Heparin dosing). Moreover, we observe that IPS$_{inv}$ outperforms IPS$_{avg}$ across multiple datasets. Consistent with the policy evaluation results, we find that NN ensemble is more effective at reducing uncertainty than BNNs. An interesting observation is that bootstrapping leads to lower rewards for warfarin datasets simulated using PHARMA logging policy. However, on further analysis, we find that this is because the PHARMA policy actions are heavily biased towards certain actions (dosage 1 in 3-action case and dosages 1 & 2 in 5-action case). This bias in the simulated actions of the logging policy leads to the learned policy being substantially biased towards action ‘1’. However, the bootstrapped framework leads to a policy which is less-biased and more balanced in its actions, although it achieves a lower overall reward. As observed in Fig. 2, policy learning using IPS$_{inv}$ achieves higher accuracy for infrequent actions (dosages 0 & 2 in 3-action and dosages 3, 4 & 5 in 5-action scenarios).

**Table 4:** Policy Learning: Rewards ($\mu \pm \sigma$) of policies learned using self-normalized inverse propensity scoring (SNIPS) formulation (10 simulations). Optdigits and Letter are two multiclass classification datasets from the UCI repository [30]. LR=Logistic Regression. NN=Neural Network

Dataset	Expert Policy/ Logging Policy	$\hat{h}_0$ - NN			
		Single NN	NN Ensemble		Adversarial
		SNIPS	SNIPS$_{inv}$	SNIPS$_{avg}$	SNIPS
UCI	OPTDIGITS (10 actions)	0.785 ± 0.113	0.800 ± 0.143	**0.805 ± 0.072**	0.767 ± 0.093
	LETTER (26 actions)	0.167 ± 0.055	0.139 ± 0.031	0.121 ± 0.047	0.157 ± 0.041
Warfarin	LR (3 actions)	0.602 ± 0.024	**0.608 ± 0.024**	0.600 ± 0.019	0.597 ± 0.024
	LR (5 actions)	0.522 ± 0.022	0.525 ± 0.021	0.527 ± 0.024	**0.532 ± 0.019**
	PHARMA (3 actions)	0.657 ± 0.015	0.646 ± 0.019	0.652 ± 0.020	**0.672 ± 0.017**
	PHARMA (5 actions)	0.602 ± 0.016	0.588 ± 0.018	0.600 ± 0.018	**0.628 ± 0.008**
Heparin	Clinician (unknown)	0.321 ± 0.030	**0.337 ± 0.046**	0.336 ± 0.054	0.325 ± 0.039

**Table 5:** Policy Learning: Rewards ($\mu \pm \sigma$) of policies learned using inverse propensity scoring (IPS) formulation (10 simulations). Optdigits and Letter are two multiclass classification datasets from the UCI repository [30]. LR=Logistic Regression. NN=Neural Network

Dataset	Expert Policy/ Logging Policy	$\hat{h}_0$ - NN			
		Single NN	NN Ensemble		Adversarial
		IPS	IPS$_{inv}$	IPS$_{avg}$	IPS
UCI	OPTDIGITS (10 actions)	0.939 ± 0.013	0.921 ± 0.035	0.940 ± 0.012	**0.942 ± 0.012**
	LETTER (26 actions)	0.429 ± 0.034	0.425 ± 0.032	0.448 ± 0.041	**0.471 ± 0.027**
Warfarin	LR (3 actions)	0.493 ± 0.040	0.506 ± 0.037	0.492 ± 0.040	**0.515 ± 0.038**
	LR (5 actions)	0.457 ± 0.034	0.469 ± 0.033	0.458 ± 0.032	**0.471 ± 0.030**
	PHARMA (3 actions)	0.656 ± 0.017	0.610 ± 0.028	0.640 ± 0.018	**0.657 ± 0.019**
	PHARMA (5 actions)	0.596 ± 0.020	0.516 ± 0.022	0.556 ± 0.020	**0.626 ± 0.012**
Heparin	Clinician (unknown)	0.295 ± 0.043	**0.317 ± 0.033**	0.311 ± 0.043	0.306 ± 0.035

### Discussions

In this study, we explored the application of policy evaluation and optimization algorithms in clinical decision-making scenarios. Acknowledging the high uncertainty in medical data and the critical need for lower risk in medical decision-making, we proposed a bootstrapping-based approach for learning decision-making policies. This method provides not only reward estimates but also confidence intervals, enabling physicians to select actions with reduced variance when necessary. Our bootstrapping framework effectively addresses the model uncertainty typically associated with IPS-based estimators, leading to decreased variance in policy evaluation and enhanced policy optimization. Furthermore, we introduced an innovative adversarial learning technique to further advance policy optimization performance. Specifically, we addressed model uncertainty from the perspective of distributionally robust counterfactual risk minimization. We proposed an adversarial IPS learner (IPS$_{adv}$), designed to maximize rewards under the worst-case propensity model within a defined uncertainty set.

Our experiments demonstrate the efficacy of our proposed frameworks (IPS*inv*, IPS*avg*, and IPS$_{adv}$) in a clinical setting involving the oral dosing of anticoagulants, heparin and warfarin. Our approaches not only facilitate better initial dosing policies but also achieve higher rewards. Moreover, we introduce the generation of semi-synthetic and real-world clinical bandit datasets to promote further research in this field. The experimental results highlight the potential of applying the policy learning paradigm to clinical applications, paving the way for various follow-up studies in this line of research. When investigating the impact of the number of bootstraps on evaluation error, we present the results of 10 simulations on the Warfarin dataset. Bootstrapping leads to improved policy learning, particularly in the PHARMA scenario, where accurately imputing the logging policy is more challenging. Furthermore, the benefits of bootstrapping are more pronounced for the IPS estimator, which is less reliable than the DR estimator for policy learning [19]. We also observe that bootstrapping using Bayesian neural networks results in lower variance compared to ensembling, owing to their enhanced ability to model the uncertainty in logged data. For both logging policies, bootstrapping both data and models yields a slight improvement over model bootstrapping alone.

### Limitations and future works

This study demonstrates the effectiveness of combining bootstrap and adversarial learning techniques in policy learning for clinical decision support. However, there are several limitations and potential avenues for future research. Firstly, our framework could be extended to incorporate other policy evaluation algorithms, especially doubly robust estimators, which may further enhance the accuracy and reliability of policy evaluation and optimization. Secondly, in real-world clinical applications, datasets often originate from multiple institutions, each with potentially different underlying distributions. Investigating methods to reduce uncertainty when learning policies from multiple real-world heterogeneous datasets would be a valuable research direction, as it could improve the generalizability and robustness of the learned policies. Lastly, personalized medicine is an increasingly important aspect of clinical decision-making. Future research should explore the development of more personalized policies that incorporate individual patient characteristics, such as comprehensive genetic information. This could lead to more tailored and effective treatment recommendations, ultimately improving patient outcomes.

## Conclusion

In this paper, we explore the application of policy evaluation and optimization algorithms in clinical decision-making scenarios. Given the significant uncertainties inherent in clinical data, our initial approach involves employing a bootstrap method to mitigate these uncertainties. Furthermore, we introduce an adversarial learning technique to enhance policy optimization performance. Our findings demonstrate the potential of leveraging the policy learning paradigm in clinical contexts, opening avenues for future research endeavors. One potential direction involves examining the integration of other policy evaluation algorithms, such as doubly robust algorithms, within our proposed framework. Additionally, there is a compelling need to explore more personalized approaches to clinical decision-making, e.g., including genetic information in the decision-making process and tailoring treatments to individual patient profiles.

## Data Availability

The real-world dataset SACHS used and/or analyzed during the current study is publicly available at (1) PharmGKB (Consortium 2009): https://www.pharmgkb.org; (2) Multiparameter Intelligent Monitoring in Intensive Care (MIMIC-III v1.4): https://mimic.mit.edu.
